# Generation and characterization of an inducible transgenic model for studying mouse esophageal biology

**DOI:** 10.1186/1471-213X-12-18

**Published:** 2012-06-12

**Authors:** Sabrina Roth, Patrick Franken, Kim Monkhorst, John Kong a San, Riccardo Fodde

**Affiliations:** 1 Department of Pathology, Josephine Nefkens Institute, PO Box 2040, 3000 CA, Rotterdam, The Netherlands; 2 Department of Cell Biology, Erasmus MC, Rotterdam, PO Box 2040, 3000 CA, Rotterdam, The Netherlands

**Keywords:** Mouse, Esophagus, Turnover rate, Promoter, ED-L2, Doxycycline inducible

## Abstract

**Background:**

To facilitate the *in vivo* study of esophageal (stem) cell biology in homeostasis and cancer, novel mouse models are necessary to elicit expression of candidate genes in a tissue-specific and inducible fashion. To this aim, we developed and studied a mouse model to allow labeling of esophageal cells with the histone 2B-GFP (H2B-GFP) fusion protein.

**Results:**

First, we generated a transgenic mouse model expressing the reverse tetracycline transactivator rtTA2-M2 under control of the promoter (ED-L2) of the Epstein-Barr virus (EBV) gene encoding the latent membrane protein-1 (*LMP-1*). The newly generated ED-L2-rtTA2-M2 (ED-L2-rtTA) mice were then bred with the previously developed tetO-HIST1H2BJ/GFP (tetO-H2B-GFP) model to assess inducibility and tissue-specificity. Expression of the H2B-GFP fusion protein was observed upon doxycycline induction but was restricted to the terminally differentiated cells above the basal cell layer. To achieve expression in the basal compartment of the esophagus, we subsequently employed a different transgenic model expressing the reverse transactivator rtTA2S-M2 under the control of the ubiquitous, methylation-free CpG island of the human hnRNPA2B1-CBX3 gene (hnRNP-rtTA). Upon doxycycline administration to the compound hnRNP-rtTA/tetO-H2B-GFP mice, near-complete labeling of all esophageal cells was achieved. Pulse-chase experiments confirmed that complete turnover of the esophageal epithelium in the adult mouse is achieved within 7–10 days.

**Conclusions:**

We show that the esophagus-specific promoter ED-L2 is expressed only in the differentiated cells above the basal layer. Moreover, we confirmed that esophageal turn-over in the adult mouse does not exceed 7–10 days.

## Background

The luminal surface of the mouse esophagus is lined by a stratified squamous epithelium, which consists of distinct zones of cell proliferation and differentiation. Tissue turnover is fueled by a population of stem cells which are thought to reside within the basal layer adjacent to the basement membrane [1]. The basal cell layer of the human esophagus is characterized by a bimodal distribution of rarely and more frequently proliferating cells [2]. The slow cycling cells appear to preferentially cluster in the interpapillary zone and were proposed, though never formally proven, to represent stem cells [2]. Recently, a subpopulation of mouse esophageal basal cells has been described as having stem-like properties such as the capacity for self-renewal and lineage-specification [3]. These cells also have the ability to extrude Hoechst dye (also referred to as ‘side population’ or SP) and are earmarked by expression of the marker CD34 [3]. The proliferating cells within the basal cell layer and the adjacent suprabasal layer may represent the analogous of transit amplifying cells in the intestinal crypt [2,4]. All basal cells can be visualized by staining for cytokeratin 14 [3]. Thus, progenitor cells arise from rare stem cell divisions, which then give rise to a population of transit amplifying cells located in the basal and suprabasal cell layers. Cell proliferation is followed by progressive cell commitment within the differentiated zone. This zone consists of multiple layers of progressively flattened and differentiated squamous cells which function as a protective barrier [4]. Differentiated cells above the basal layer can be visualized by cytokeratins 4 and 13 (CK4 and CK13) [3].

Because of their rapid and alarming increase during the last 30–40 years, esophageal malignancies have become the 6^th^ leading cause of cancer death worldwide [5]. Two major forms of esophageal cancer are known: squamous cell carcinoma and esophageal adenocarcinoma (EA). EA is typically developing in patients affected by Barrett’s esophagus, a premalignant condition characterized by the progressive replacement of the normal stratified squamous epithelium of the lower esophagus with columnar cells of the intestinal type [6]. Barrett’s esophagus is thought to arise as a consequence of gastro-esophageal reflux disease and the exposure of the esophageal epithelium to gastro-duodenal juices causing tissue damage and inflammation. However, the molecular and cellular mechanisms underlying adenocarcinoma and squamous cell carcinoma in the esophageal epithelium are yet largely unknown. In part, this is due to the lack of *in vivo* experimental tools to study the esophageal stem cell niche during homeostasis and its role in the genesis of malignancies of the squamous and adenocarcinoma type. Many genes have been implicated in esophageal tumorigenesis. The temporal and spatial control over expression of specific genes can be achieved through the use of tissue-specific and inducible transgenic mouse models [7]. To date, the only promoter described in the literature to be expressed at high levels in the esophagus is encompassed within a 0.6 kb ED-L2 fragment from the 3’ non-coding sequence of the gene encoding the latent membrane protein-1 (*LMP-1*) of the Epstein-Bar virus (EBV) [8,9]. The expression of this promoter has been carefully characterized and is restricted specifically to the tongue, pharynx and esophagus [8]. Hence, we first employed the ED-L2 promoter to generate a tetracycline-inducible mouse model allowing esophagus-specific gene expression.

## Results

### Generation of transgenic ED-L2-rtTA mice

**Figure 1:**
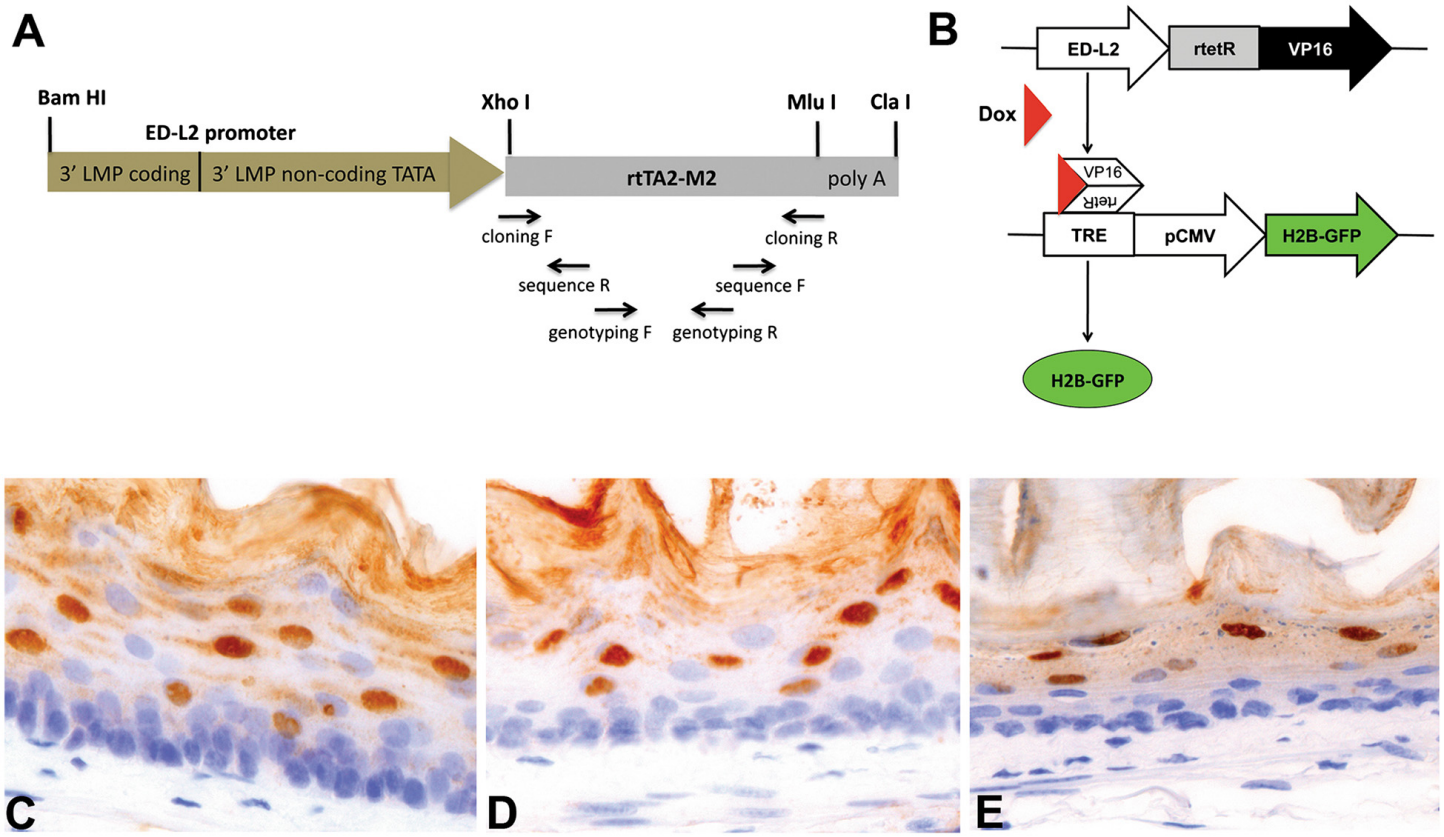
**“Tet On” regulated expression of the H2B-GFP fusion protein in the adult mouse esophagus.A**) Schematic map of the ED-L2-rtTA fragment here developed and employed for transgenic mouse generation. **B**) Following doxycycline pulse, compound heterozygous ED-L2-rtTA/tetO-H2B-GFP transgenic animals express H2B-GFP. **C**-**E**) IHC analysis of H2B-GFP expression. After 7 days of doxycycline treatment (pulse), compound ED-L2-rtTA/tetO-H2B-GFP transgenic mice from all three founder lines (**C**. line 1; **D**. line 5, **E**. line 6, all 40x magnification) show strong nuclear H2B-GFP expression.

**Figure 2:**
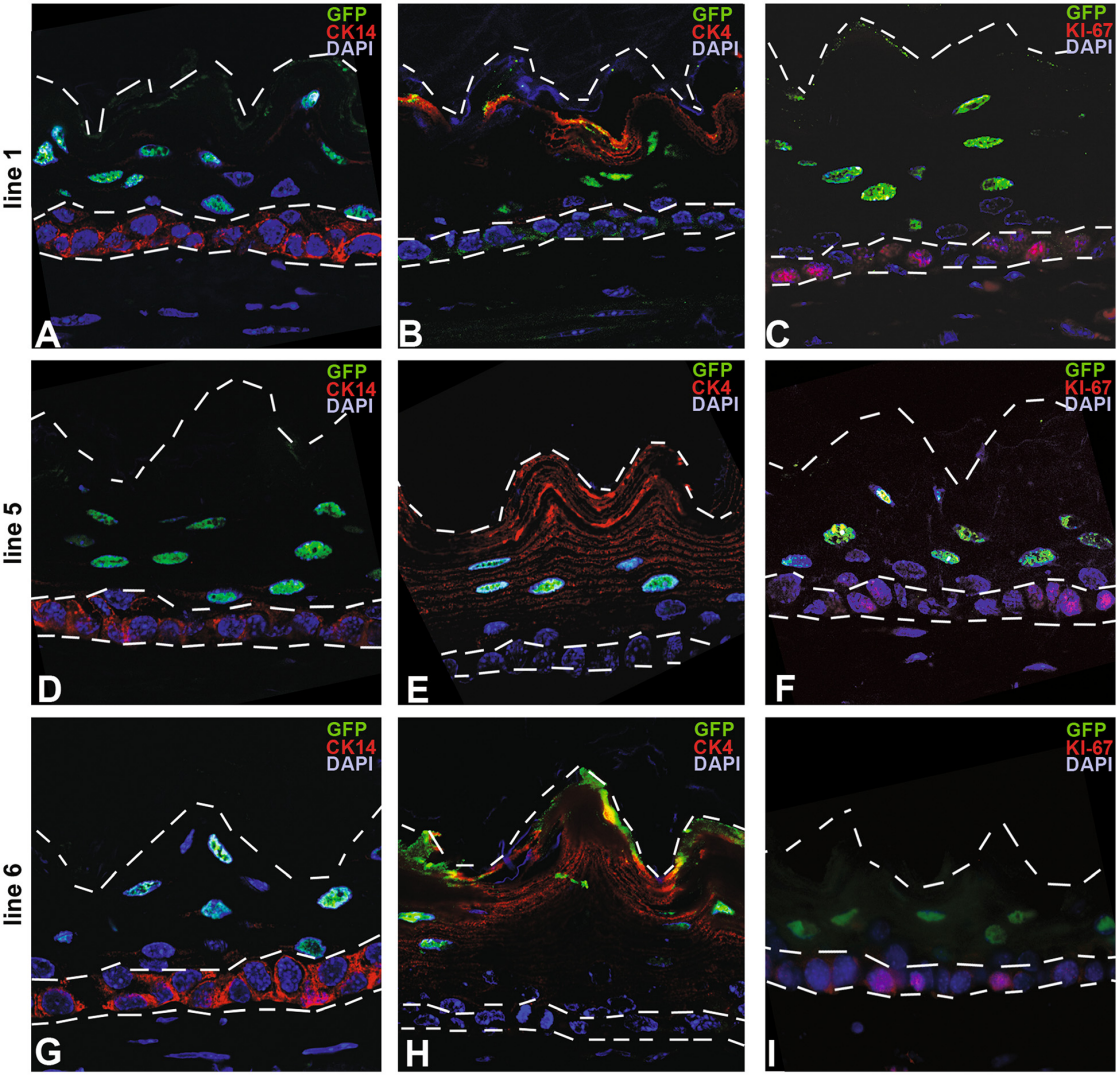
**H2B-GFP expressing cells are located exclusively within the differentiated non-basal cell layers.** Immunofluorescence double staining for GFP (green, nuclear) and **A**,**D**,**G**) CK14 (red, basal cell layer), **B**,**E**,**H**) CK4 and **C**,**F**,**I**) CK13 (both red, non-basal cell layers) in all three founder lines (**A**-**C** line 1; **D**-**F** line 5, **G**-**I** line 6, all 40x magnification). The dashed lines indicate the outline of the esophageal epithelium and the border to the basal cell layer.

Transgenic ED-L2-rtTA founder lines were generated with a construct consisting of the ED-L2 promoter cloned in front of the reverse tetracycline transactivator rtTA2-M2, previously described as an improved rtTA variant (‘Tet-On’) with strongly reduced background activity and a 10-fold increased sensitivity for doxycycline-driven induction [10] (Figure 1A). PCR-analysis identified six founder lines 5 of which showed transmission to the germline. Functionality of the transgene was tested by breeding the transgenic animals with tetO-H2B-GFP mice expressing the histone H2B-green fluorescent protein (H2B-GFP) under the control of a tetracycline-responsive regulatory element [11]. Compound ED-L2-rtTA/tetO-H2B-GFP transgenic animals were administered drinking water supplemented with 5% sucrose and 2 mg/ml doxycycline (Figure 1B). Induction of H2B-GFP gene expression was analyzed by immunohistochemistry (Figure 1C-E) and fluorescence microscopy (Figure 2). Three transgenic lines were identified that showed homogeneous and high H2B-GFP expression levels throughout the entire length of the esophagus. H2B-GFP expression in all three independent founder lines appeared to be restricted to the compartment of differentiated cells above the basal layer though not in the basal layer (Figure 1C-E).

### Detailed analysis of ED-L2-rtTA transgene expression

**Figure 3:**
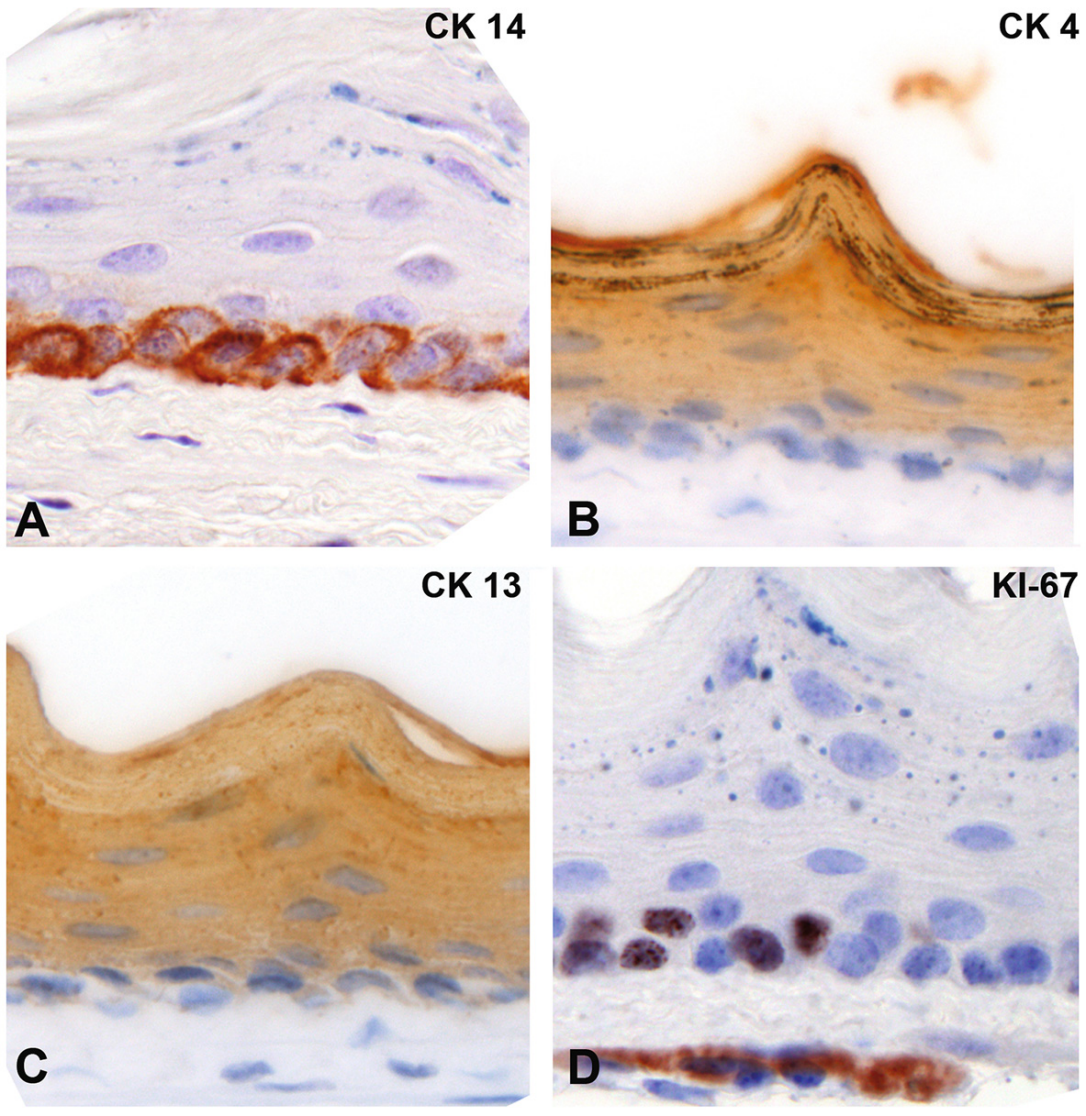
**IHC analysis of the squamous epithelium of the mouse esophagus.** Immunohistochemistry for cytokeratin 14 marks the basal layer (**A**), while cytokeratin 4 and 13 are expressed in the differentiated cell layers above the basal cells (**B**, **C**). The proliferation marker Ki-67 is expressed in distinct basal cells (**D**). All images were taken at 40x magnification.

To analyze in more detail, which cell layers of the esophagus showed expression of H2B-GFP upon doxycycline-induction, we performed double staining for GFP and basal as well as non-basal cytokeratins and the proliferation marker Ki-67. While CK14 is a marker of all basal cells (Figure 3A), CK4 and CK13 are highly expressed in suprabasal, spinous and granular cells (Figure 3B-C), whereas Ki-67 expression is observed in a subset of the basal cells (Figure 3D) [3]. Staining for H2B-GFP and the basal markers CK14 (Figure 3A,D,G) and Ki-67 (Figure 2C,F,I) was never observed in the same cell. However, GFP and CK4 (Figure 2B,E,H) were coexpressed. Thus, H2B-GFP expressing cells are located within the differentiated cell layers above the basal cell layer. Cells within the basal cell compartment are devoid of H2B-GFP expression.

### Compound hnRNP-rtTA mice allow complete labeling of esophageal cells

**Figure 4:**
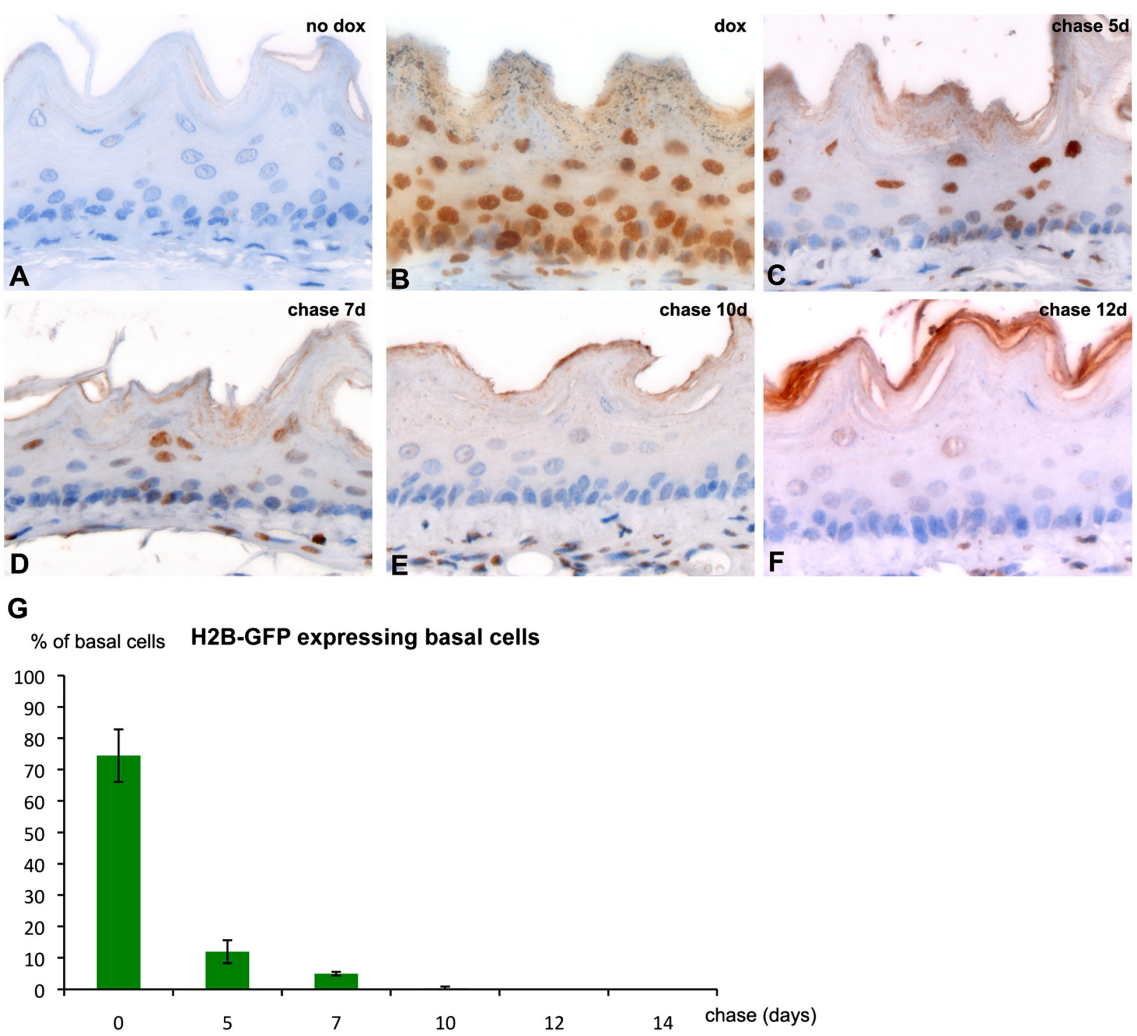
**IHC analysis of H2B-GFP expression in pulse-chase experiments with compound hnRNP-rtTA/tetO-H2B-GFP mice.A**) Untreated compound animals do not express nuclear H2B-GFP. **B**) After 7 days of doxycycline treatment in the drinking water (pulse) the vast majority of esophageal cells are positive for nuclear GFP expression. After the doxyxycline pulse, mice were chased (no dox in drinking water) and analyzed at 5 (**C**), 7 (**D**), 10 (**E**) and 12 days (**F**). Images were taken at 60x magnification. (**G**) The average number of H2B-GFP expressing basal cells of animals chased for 0, 5, 7, 10, 12 and 14 days is displayed. Displayed are the average percentages of H2B-GFP expressing cells from the total population of basal esophageal cells (±standard deviation).

To achieve a broader expression pattern in the various cell layers of the esophagus, we subsequently employed a different transgenic model expressing the reverse transactivator rtTA2S-M2 under the control of the ubiquitous and methylation-free CpG island of the human hnRNPA2B1-CBX3 gene [12]. Exclusively upon doxycycline administration to the compound hnRNP-rtTA/tetO-H2B-GFP mice, near-complete labeling of esophageal cells was achieved (Figure 4A-B). The observation that the vast majority of cells are labeled upon doxycycline administration allowed us to perform pulse-chase experiments to study the dynamics of cell turnover in the esophagus and the alleged presence of infrequently dividing, label-retaining cells (LRCs). To this aim, we withdrew doxycycline from the drinking water of previously pulsed hnRNP-rtTA/tetO-H2B-GFP mice and analyzed their esophagi by GFP IHC at different time points. As shown in Figure 4C-F and 4G, progressive dilution of H2B-GFP^+^ cells was observed, with suprabasal and more differentiated squamous cells persisting up to 7 days (Figure 4D). This is in agreement with previous studies showing a complete turnover of the human esophageal epithelium within 4–8 days [13]. As shown in Figure 4, upon doxycycline pulse, 83% of all cells of the basal layer were marked with nuclear H2B-GFP. Following doxycycline withdrawal, LRCs were observed for no longer than 7 days within the basal cell layer (Figure 4G).

## Discussion

This study shows that the ED-L2 promoter is not active in the basal cell layer of the mouse esophagus where stem cells are thought to reside. For the purpose of studying the mechanisms of esophageal cancer onset and progression, it is of utmost importance to drive expression of candidate genes to distinct cell types located within the basal cell layer. Overexpression of the human cyclin D1 oncogene under control of the ED-L2 promoter resulted in the development of dysplasia with late onset (16 months) [9]. Similarly, *Klf5*-overexpression gated by the same ED-L2 promoter results in a two-fold increase in the proliferation of basal cells [14]. The mild hyper- and dysplastic phenotypes observed in these models suggest that ED-L2-driven oncogene (over)expression is insufficient to promote full-blown tumor formation in the esophagus. This is likely to be due to the non-basal expression pattern of the viral promoter, i.e. in a compartment where progenitor but not stem cells are thought to reside [2,4]. In the intestine, loss of function mutations at the *Apc* tumor suppressor gene have been shown to trigger tumor formation exclusively from cycling stem cellsthough not from progenitor, transient amplifying cells [15]. Likewise, it is plausible that the same holds true for the esophagus where oncogene expression under the non-basal ED-L2 promoter is insufficient for tumor formation. The ED-L2-rtTA mouse model is of potential use in such proof-of-principle studies and can be employed as an example of oncogene expression in non-basal esophageal cells. However, an esophageal promoter, which is specifically active within the stem cell compartment, has still to be found in future experiments.

Novel esophageal-specific promoters are needed to study the role played by resident stem cells in homeostasis and cancer. As shown here, a more ubiquitous promoter can be employed to drive expression of the H2B-GFP marker protein within the esophagus. However, as described by Katsantoni et al., this promoter is widely expressed throughout the body in organs such as the kidney, heart, liver, lung and spleen [12]. So while this promoter can be useful to study esophageal biology, it is not applicable to specifically overexpress oncogenes within the esophagus only. We have employed the ubiquitous promoter in a pulse chase study and confirmed that esophageal turn-over in the adult mouse does not exceed 7–10 days. Notably, quiescent LRCs were detected for no longer than 7 days within the esophageal epithelium. This observation is suggestive of the cycling nature of the resident stem cells of the mouse esophagus. However, since complete labeling of all esophageal basal cells after pulse was not achieved (83%, Figure 4G), it is still feasible that the ubiquitous promoter is specifically silenced within the quiescent, and thus potentially label-retaining esophageal cells.

## Conclusion

Future studies should be directed to the identification of esophageal stem cell-specific genes and their promoters to allow lineage tracing and the introduction of specific gene mutations to assess their potential as cell of origin of esophageal cancer.

## Methods

### Transgenic mice

All animal experiments were approved by the DEC commission and performed according to institutional and national regulations. Phenotypic analysis was performed in both male and female animals of mixed (C57BL6/J and CD1) genetic background. All animals were fed ad libidum and housed in SPF facilities. The ED-L2-rtTA2-M2 construct was generated as follows. The rtTA2-M2 sequence, was PCR-amplified from the pUHrT62-1 vector [10] (gift from W. Hillen, Erlangen) using the cloning forward (5’-ATTCTCGAGGCCGCCACCATGTCTAGACTG-3′) and the cloning reverse (5′-TAGACGCGTTTATCCTGGGAGCATGTCAAGG-3′) primers (Figure 1A). The PCR product was then subcloned downstream of the ED-L2 promoter [8] (ED-L2 vector kind gift from Joanna B. Wilson, Glasgow) by digesting both PCR product and vector with the restriction enzymes XhoI and MluI, and ligating both fragments. The final vector was sequence verified using the sequencing pri-mers 5′-CTTTTCGGCCTGGAACTAATC-3′ and 5′-CTGTCCAGCATCTCGATTG-3′; (Figure 1A). The 2.1 kb expression cassette was cut out of the plasmid backbone by BamHI and ClaI digest, gel-purified and prepared for injection into fertilized C57BL6/J oocytes. Founders were identified by PCR amplification of tail DNA using transgene specific genotyping primers (5′-CAAGACTTTCTGCGGAACAAC-3′ and 5′-GTGTCTCTCTTTCCTCTTTTG-3′, Figure 1A). Transgenic ED-L2-rtTA animals (C57BL6/J) were bred with tetO-H2B-GFP animals (CD1) [11] (kindly provided by E. Fuchs, New York). Transgene expression was induced in compound heterozygous animals and their littermates by replacing normal drinking water with 5% sucrose water containing 2 mg/ml doxycycline (Sigma, D9891, Figure 1B). Dox-treated water was changed every 2 days. After 7 days of doxycycline treatment, mice were sacrificed and tissues were analysed for H2B-GFP-expression. The same pulse scheme was also applied to the compound hnRNP-rtTA/tetO-H2B-GFP mice. After one week, pulsed mice were withdrawn doxycycline from the drinking water and sacrificed at 0, 5, 7 and 10 days (chase).

### Immunohistochemistry

Tissues were fixed in 4% PFA and embedded in paraffin. Four μm sections were mounted on slides and stained by HE for routine histology. Immunohistochemistry was performed according to standard procedures using the following antibodies: GFP (1:800, A11222, Invitrogen), CK4 (1:100, ab11215, Abcam), CK13 (1:100, ab16112, Abcam), CK14 (1:10000, PRB-155B, Covance) and Ki-67 (1:50, M7249, DAKO). Signal detection was performed using Rabbit EnVision + System-HRP (K4011, Dako) for GFP. CK4 and CK13 were detected using Goat-anti-Mouse-HRP, CK14 using Goat-anti-Rabbit-HRP. The average number of H2B-GFP expressing basal cells was determined by counting ca. 300 cells in three different sections of the esophagi of animals chased for 0, 5, 7, 10, 12 and 14 days. Displayed are the average percentages of H2B-GFP expressing cells from the total population of basal esophageal cells (±standard deviation).

### Immunofluorescence

GFP (1:50, A11222, Invitrogen), Ki-67 (1:50, M7249, Dako), CK4 (1:100, BD Pharmingen) ab11215, Abcam), CK13 (1:100, ab16112, Abcam) and CK14 (1:1000, PRB-155B, Covance) were employed for Immunofluorescence analysis. Ki-67 was detected using rabbit-anti-rat-A594 (Invitrogen), CK4 and CK13 were detected using goat-anti-mouse-A594 (Invitrogen) and goat-anti-rabbit-A488 (Invitrogen) was used for signal detection of GFP.

## Abbreviations

CK, Cytokeratin; EA, Esophageal adenocarcinomas; EBV, Eppstein-Barr Virus; H2B-GFP, Histone H2B-green fluorescent protein; LMP-1, Latent membrane protein-1; LRCs, Label-retaining cells.

## **Competing interests**

The authors declare that they have no competing interests.

## Authors’ contributions

SR and PF performed the mouse analysis and the stainings. KM cloned the ED-L2-rtTA construct. JKAS performed the oocyte-injection. SR and RF wrote the paper. RF designed the study. All authors read and approved the final manuscript.
